# SARS Antibody Test for Serosurveillance

**DOI:** 10.3201/eid1009.040101

**Published:** 2004-09

**Authors:** Po-Ren Hsueh, Chuan-Liang Kao, Chun-Nan Lee, Li-Kuan Chen, Mei-Shang Ho, Charles Sia, Xin De Fang, Shugene Lynn, Tseng Yuan Chang, Shi Kau Liu, Alan M. Walfield, Chang Yi Wang

**Affiliations:** * National Taiwan University College of Medicine, Taipei, Taiwan;; † Tsu Chi University, Hualien, Taiwan;; ‡ Academia Sinica, Taipei, Taiwan;; § United Biomedical, Inc., Hauppauge, New York, USA;; ¶ United Biomedical, Inc. Asia, Hsin Chu, Taiwan

**Keywords:** research, SARS-CoV, serologic test, enzyme-linked immunosorbent assay, synthetic peptide, S protein, M protein, N protein, specificity, sensitivity, retrospective study

## Abstract

A peptide-based enzyme-linked immunosorbent assay (ELISA) can be used for retrospective serosurveillance of severe acute respiratory syndrome (SARS) by helping identify undetected chains of disease transmission. The assay was developed by epitope mapping, using synthetic peptides from the spike, membrane, and nucleocapsid protein sequences of SARS-associated coronavirus. The new peptide ELISA consistently detected seroconversion by week 2 of onset of fever, and seropositivity remained through day 100. Specificity was 100% on normal blood donor samples, on serum samples associated with infection by other pathogens, and on an interference panel. The peptide-based test has advantages of safety, standardization, and automation over previous immunoassays for SARS. The assay was used for a retrospective survey of healthy healthcare workers in Taiwan who treated SARS patients. Asymptomatic seroconversions were detected in two hospitals that had nosocomial disease.

## Abstract 2

A standardized and rapid peptide-based SARS assay is characterized for sensitivity and specificity.

Retrospective surveillance for infection is an important means to screen for and interrupt undetected chains of disease transmission. Such surveillance may be key to tracking the severe acute respiratory syndrome–associated coronavirus (SARS-CoV) because mild and asymptomatic cases of SARS-CoV infection that do not meet the World Health Organization’s case definition (*1*) have been identified by immunoassays (*2**–**4*), and SARS-CoV–like viruses have been isolated from wild mammals (*5*). SARS-CoV may have persisted over the summer in previously affected areas in such difficult-to-recognize reservoirs (*6*). The reemergence of SARS in the city of Guangzhou of the Guangdong Province of China in December 2003 and January 2004 (*7*) is evidence that an unknown reservoir exists and signals the need for continued surveillance with laboratory testing.

The current laboratory methods for identifying SARS are not ideal tools for use in retrospective mass screening. Reverse transcription–polymerase chain reaction (RT-PCR) for detecting viral RNA is the most sensitive method for early identification of SARS. However, viral load rapidly declines beginning 9 or 10 days after disease onset (*8**–**10*). Moreover, RT-PCR requires sophisticated equipment and high laboratory quality-assurance standards (*11**,**12*). Identifying seroconversion to SARS-CoV by immunoassay also is a definitive criterion for laboratory determination of SARS (*13*), and seroconversion is the preferred standard for retrospectively detecting SARS-CoV infection (*14*). SARS immunoassays include the enzyme-linked immunosorbent assay (ELISA) or Western blot with antigen from whole virus or various recombinant proteins, a cumbersome immunofluorescence assay (IFA) using whole virus fixed on glass, and methods to determine neutralizing antibodies (*10**,**11*). Immunoglobulin (Ig) G to SARS-CoV, detected by these immunoassays, begins to rise sharply by day 11 after onset of symptoms. Virtually all SARS patients show virus-specific antibody by week 3, and anti–SARS-CoV IgG persists through day 100 (*8**,**10**,**15*). Although any of these immunoassays can provide a definitive laboratory finding, all but the recombinant tests require biosafety level 3 to contain the virus or are time-consuming to perform, have not been well-standardized, are of unknown specificities, and would be difficult to adapt to large-scale manufacture. Improving laboratory methods for the large-scale serologic surveillance of SARS, particularly in the presence of other respiratory illnesses, and standardization of diagnostic assays are key priorities for controlling SARS (*16*). In this report, a standardized and rapid peptide-based SARS ELISA is characterized for sensitivity and specificity.

Beginning in April 2003, delay in recognizing SARS cases and in implementing isolation procedures led to several nosocomial clusters of SARS-CoV transmission in healthcare facilities in Taiwan (*17**,**18*). The results from a retrospective serologic survey by the peptide ELISA of healthcare workers from facilities affected by nosocomial outbreaks are presented as a working example.

## Materials and Methods

### Synthetic Peptide ELISA

**Figure 1:**
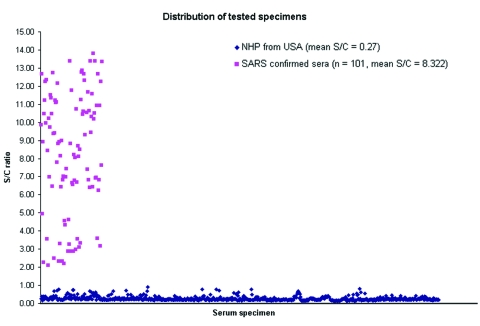
Distribution of tested specimens. Signal/cutoff (S/C) distribution of Center for Disease Control (Taiwan) SARS (severe acute respiratory syndrome) serum panel and blood donor serum panel. The mean signal/cutoff (S/C) ratio for the SARS samples was 8.08. The mean S/C for the 1,390 normal human plasma was 0.28.

We developed an ELISA for SARS that has synthetic peptide antigens as the solid-phase immunosorbent. Over 200 overlapping peptides, deduced from the Tor2 SARS-CoV genomic sequence (*19*), were synthesized as candidate antigens from the spike (S), membrane (M), and nucleocapsid (N) proteins. Candidate immunodominant S, M, and N peptides were selected and refined on the basis of serologic reactivities to a panel of serum samples from 13 patients clinically diagnosed with SARS at National Taiwan University Hospital in Taipei and the Xiaotangshan SARS Emergency Hospital in Beijing (*20*). Epitope mapping by serologic validation has been described for the development of peptide-based ELISA tests for HIV, hepatitis C virus, and foot-and-mouth disease virus (*21**–**23*). For the peptide-based SARS ELISA, wells of microtiter plates were coated with 2 µg/mL of a mixture of the S, M, and N protein–derived peptides, and serologic reactivities were determined by a standard ELISA procedure as previously described (*23*), except that the detector was horseradish peroxidase–conjugated goat anti-human IgG, and the chromaphore was 3,3´,5,5´-tetramethylbenzidine (TMB). In brief, serum samples, including two normal human samples provided as nonreactive controls, were diluted 1:20 in phosphate-buffered saline with carrier proteins and preservative. The diluted serum samples were reacted to the peptide-coated microtiter wells for l h at 37°C. Plates were washed 6 times, reacted to the antibody conjugate, again washed 6 times, and reacted to TMB; reactivity was then determined by A_450_. Assay results were obtained within 3 h. Results were scored on the basis of the signal/cutoff (S/C) ratio, and cutoff absorbance was determined from the mean of the two controls plus 6 standard deviations (SD) from the distribution of normal human samples (Figure 1).

### Serum Panels

Seroconversion panels were collected as serial serum samples from SARS patients at National Taiwan University Hospital in the course of treatment. These panels tested positive for seroconversion by IFA (*24*) and are used here to evaluate analytical sensitivity.

A panel of 69 serum samples from convalescent SARS patients were provided by the Center for Disease Control, Department of Health, Taiwan, to evaluate diagnostic sensitivity. These serum specimens were confirmed for SARS-CoV infection clinically by the World Health Organization diagnostic criteria (*1*) and serologically by whole virus–based ELISA and IFA; some specimens were also confirmed by RT-PCR (*24*). Samples were drawn with appropriate timing for serologic reactivity (7–25 weeks after onset of symptoms). A panel of 1,390 plasma samples collected from random blood donors in Florida before 2001 was obtained from the Gulf Coast Regional Blood Center (Houston, TX) for specificity evaluation.

Additional specificity studies were conducted with serum that had serologic reactivities for bloodborne pathogens (HIV-1, HIV-2, hepatitis C virus, HTLV 1/II, and syphilis) obtained by United Biomedical Inc. before 2000 from various U.S. sources, an interference panel supplied by Boston Biomedica Inc. (Boston, MA) of serum samples with interference substances commonly found in processed clinical samples (EDTA, acid citrate dextrose, and citrate phosphate dextrose with adenine), and serum supplied by National Taiwan University Hospital from patients associated with typical and atypical respiratory pathogens other than SARS-CoV (influenza, rubella, cytomegalovirus, Epstein-Barr virus, and *Mycoplasma pneumoniae*). Serum samples were collected from healthy healthcare workers after interviews to confirm lack of signs and symptoms of SARS including fever, respiratory symptoms, and diarrhea.

## Results

### Sensitivity

**Figure 2:**
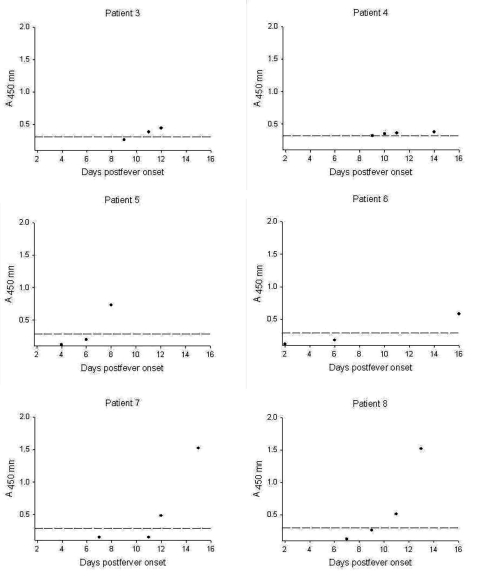
Time of seroconversion from onset of fever for patients infected with severe acute respiratory syndrome–associated coronoavirus. Cutoff absorbance shown by dotted line.

The peptide-based ELISA was evaluated for sensitivity to seroconversion on eight seroconversion panels obtained from National Taiwan University Hospital (Figure 2, Table 1). In patient 1, seroconversion was detected by day 11 with an A_450_ of 1.638. Absorbance remained at >2 at day 97. In patient 2, from whom blood was drawn on days 0, 6, 16, 27, and 116 (no samples were collected from day 6 to day 16), seroconversion was apparent on day 16 after the onset of fever. On day 116, the A_450_ remained >3. In five of the other six seroconversion panels, from acute to convalescent phases, seropositivity was observed by days 8 to 12, and by day 16 in patient 6, from whom serum had not been collected from days 6 to 16 (Figure 2). The peptide-based ELISA showed an analytical sensitivity to earliest time of detection by week 2 and for duration of detection beyond day 100. The seroreactivities of patients 3 to 8 were also evaluated by a standard IFA method (*24*) for comparison. Seroconversion was detected in all six patients by the IFA method within 2 days of the peptide ELISA (data not shown).

**Table 1:** Sensitivity of peptide-based SARS-CoV ELISA from two patients at NTU^a^

Patient/day	A_450nm_	S/C ratio
SARS patient 1		
Day 0	0.119	0.44
Day 11	1.638	6.09
Day 17	2.447	9.10
Day 38	2.749	10.22
Day 97	2.600	9.67
SARS patient 2		
Day 0	0.068	0.25
Day 6	0.163	0.61
Day 16	0.345	1.28
Day 27	1.212	4.51
Day 116	>3.000	>9.40

^a^SARS, severe acute respiratory syndrome; CoV, coronavirus; ELISA, enzyme-linked immunosorbent assay; S/C = signal/cutoff ratio; NTU, National Taiwan University.

The diagnostic sensitivity of the peptide ELISA was 100% on a panel of 69 convalescent-phase serum samples from SARS patients provided as a reference panel by the Center for Disease Control, Department of Health, Taiwan. These sera, confirmed for SARS by diagnostic and serologic criteria, displayed a mean S/C ratio of 8.08 (Figure 1).

### Specificity

The specificity of the peptide-based SARS ELISA was tested on plasma obtained before 2001 from 1,390 random Florida blood donors (Gulf Coast Regional Blood Center, Houston, TX). These normal plasma samples with a presumed zero seroprevalence rate gave a mean A_450_ of 0.074 ± 0.0342 (SD). Subsequently, the cutoff value for the peptide-based assay was set as the mean A_450_ for duplicate nonreactive controls plus 0.279 (based on 6 SDs from the mean for these 1,390 normal plasma samples). The distribution of the S/C ratio for the blood bank samples is plotted in Figure 1 with the mean S/C ratio of 0.28. None showed positive reactivity, for a specificity on the normal samples of 100% (Table 2).

**Table 2:** Sensitivity and specificity of peptide-based ELISA^a^

Source of samples	Total no.	ELISA+	ELISA–
Blood donors (Gulf Coast Regional Blood Bank, USA)	1,390	0	1,390
Blood-transmitted pathogen panel (various blood banks, USA)	52	0	52
Interference panel (BBI)	41	0	41
Confirmed SARS (TW CDC)	69	69	0
Influenza patients (NTU)	10	0	10
Influenza vaccinees (NTU)	16	0	16
Rubella patients (NTU)	10	0	10
EBV patients (NTU)	9	0	9
*Mycoplasma* (NTU)	5	0	5
CMV patients (NTU)	8	0	8

^a^ELISA, enzyme-linked immunosorbent assay; NTU, National Taiwan University; BBI, Boston Biomedica Inc (Boston, MA); TW CDC, Taiwan Center for Disease Control; CMV, cytomegalovirus; EBV, Epstein-Barr virus.

The peptide ELISA was further evaluated for specificity with a pre-2000 collection of serum samples from patients with seropositivities for bloodborne pathogens such as HIV-1, HIV 2, hepatitis C virus, HTLV 1/II, and syphilis (various U.S. sources), and with normal serum samples spiked with interference substances heparin, EDTA, ACD, and CPDA-1 (Boston Biomedica). The 52 samples with seroreactivities for the pathogens all tested negative by the peptide-based SARS ELISA, as did the 41-sera interference panel (Table 2).

The peptide ELISA was evaluated for specificity on serum samples drawn from patients associated with typical and atypical respiratory pathogens other than SARS-CoV (National Taiwan University Hospital). These included samples from 1) 10 patients naturally infected with influenza (two sequential bleeds per influenza patient), 2) 10 patients with rubella, 3) 8 patients with cytomegalovirus infection, 4) 9 patients with Epstein-Barr virus, 5) 5 patients infected with *Mycoplasma pneumoniae*, a bacterial agent for atypical pneumonia, and 6) pre- and postvaccine blood samples from 16 patients given influenza vaccine. All samples were tested in duplicate. The site-specific antigens of the peptide SARS-CoV ELISA were free of cross-reactivities to the other respiratory pathogens (Table 2).

### Serologic Survey of Healthcare Workers

A prospective study was performed to determine asymptomatic infection among primary healthcare workers in hospitals that treated SARS patients. We collected serum samples from 623 healthcare workers without symptoms, not all of whom were in direct contact with SARS patients, who agreed to be tested for antibody to SARS-CoV at Ho Ping, Yang Ming, En Chu Kong, and Hsin Tai Hospitals, approximately 4 weeks after the outbreaks were recognized. Ho Ping and Yang Ming Hospitals had admitted SARS patients before the recognition of SARS and before healthcare workers had implemented control measures. Subsequently, these facilities experienced transmission to healthcare workers. The En Chu Kong and Hsin Tai facilities admitted patients once control measures had been implemented. Neither of these hospitals recorded transmission of SARS to healthcare workers.

ELISA detected three cases out of 383 samples from Ho Ping and one case in 50 blood samples from nursing aides at Yang Ming. These four positive samples, indicative of asymptomatic infection, were confirmed for seropositivity by IFA. None of the 190 serum samples from the two hospitals without nosocomial infection displayed seroconversion.

## Discussion

A convenient ELISA to detect IgG to SARS-CoV, based on site-specific synthetic antigens taken from the S, M, and N proteins of the virus, has high specificity. No cross-reactivity was detected in samples associated with common non-coronavirus respiratory pathogens. In addition, the lack of detectable reactivities among the 1,390 U.S. blood donors supports a specificity for the assay to distinguish SARS-CoV infection from infection by other human coronaviruses. The presence of anti-coronavirus antibodies among a U.S. population of this size is strongly anticipated because an incidence as high as 8% for OC43 and 229E respiratory infections has been observed, even among healthy young adults (*25*).

The new peptide-based ELISA is equivalent in sensitivity to other immunoassays for SARS and can be detected after day 100. The synthetic antigens provide the advantages of high standardization, freedom from biohazard, and ease of scale-up production. Moreover, testing by the ELISA format can be readily automated for large-scale screening. The highly specific peptide-based SARS antibody test is a convenient means to carry out widespread retrospective surveillance, such as that now being proposed for China to trace hotspots of persons carrying antibodies to SARS-CoV and to track the origins of the disease (*26*).

A preliminary survey with the peptide ELISA detected asymptomatic clusters of seroconversion among exposed healthcare workers in two Taiwan hospitals that also had nosocomial disease. In contrast, no seroconversion was found among the exposed healthcare workers from two hospitals that had no apparent disease transmission to healthcare workers. The finding of asymptomatic seropositive persons indicates that the test will be useful in larger retrospective surveillance studies, which are needed to fully define the epidemiology and spectrum of disease.
